# An increase in glycoprotein concentration on extracellular virions dramatically alters vaccinia virus infectivity and pathogenesis without impacting immunogenicity

**DOI:** 10.1371/journal.ppat.1010177

**Published:** 2021-12-28

**Authors:** Stephanie R. Monticelli, Peter Bryk, Matthew G. Brewer, Hector C. Aguilar, Christopher C. Norbury, Brian M. Ward

**Affiliations:** 1 Department of Microbiology and Immunology, University of Rochester Medical Center, Rochester, New York, United States of America; 2 Department of Dermatology, University of Rochester Medical Center, Rochester, New York, United States of America; 3 Department of Microbiology and Immunology, Cornell University, Ithaca, New York, United States of America; 4 Department of Microbiology and Immunology, The Pennsylvania State University College of Medicine, Hershey, Pennsylvania, United States of America; The Australian National University, AUSTRALIA

## Abstract

The extracellular virion (EV) form of Orthopoxviruses is required for cell-to-cell spread and pathogenesis, and is the target of neutralizing antibodies in the protective immune response. EV have a double envelope that contains several unique proteins that are involved in its intracellular envelopment and/or subsequent infectivity. One of these, F13, is involved in both EV formation and infectivity. Here, we report that replacement of vaccinia virus F13L with the molluscum contagiosum virus homolog, MC021L, results in the production of EV particles with significantly increased levels of EV glycoproteins, which correlate with a small plaque phenotype. Using a novel fluorescence-activated virion sorting assay to isolate EV populations based on glycoprotein content we determine that EV containing either higher or lower levels of glycoproteins are less infectious, suggesting that there is an optimal concentration of glycoproteins in the outer envelope that is required for maximal infectivity of EV. This optimal glycoprotein concentration was required for lethality and induction of pathology in a cutaneous model of animal infection, but was not required for induction of a protective immune response. Therefore, our results demonstrate that there is a sensitive balance between glycoprotein incorporation, infectivity, and pathogenesis, and that manipulation of EV glycoprotein levels can produce vaccine vectors in which pathologic side effects are attenuated without a marked diminution in induction of protective immunity.

## Author summary

Viral glycoproteins are critical determinants of host cell tropism, immunity, and pathogenesis. Vaccinia virus was used for the most successful immunization program in history, and poxviruses continue to be used as vaccine vectors. Here, we report that vaccinia virus extracellular virion (EV) protein F13 plays an important, previously unappreciated, role in controlling glycoprotein incorporation, and that there is a direct relationship between glycoprotein concentrations and subsequent infectivity. Crucially, manipulation of the EV glycoprotein concentrations altered pathogenesis and lethality in an in vivo infection model, but did not markedly alter the induced immune response. These results have important implications that inform the design of safer and more efficacious poxvirus-based vaccine vectors by altering glycoprotein content.

## Introduction

Viral glycoproteins are a major component of the outermost envelope of viruses, actively participating in critical aspects of the viral lifecycle [1,2]. The interaction between viruses and their hosts is often determined by interactions between viral glycoproteins and host cell receptors, and thus deletion/mutation of glycoproteins found in the envelope of viruses typically impact host cell entry, host range, and pathogen recognition. As such, viral glycoproteins are a major target of neutralizing antibodies [3–8]. This combination of factors, in turn, affects viral pathogenesis [1,2,9].

Vaccinia virus (VACV), the prototypical member of the *Orthopoxvirus* genus, produces two morphologically and antigenically distinct infectious forms of virions during its replication cycle: intracellular mature virions (IMV) and extracellular virions (EV) [10]. Following IMV production, a subset are trafficked to the *trans-*Golgi network, where two additional membranes are added to produce intracellular enveloped virions (IEV). IEV are a transient form that are transported to the cell surface, where fusion with the plasma membrane releases the EV form of the virus, which is critical for cell-to-cell spread and long-range dissemination [11–15]. Both IMV and EV are surrounded by lipid envelopes, but the EV surface proteins are glycosylated whereas the IMV surface proteins are not. Four proteins found in EV and not IMV (A33, A34, B5 and F13), are highly conserved between members of the *Orthopoxvirus* genus [16–21]. These proteins play critical roles in the formation of infectious EV as deletion of any one of them results in a small plaque phenotype [22–25]. Of these four, deletion of F13 has the most profound effect on EV production due to defects in both EV production and infectivity [26,27].

To gain a greater insight into the molecular function of F13, a recombinant VACV was created that replaced F13L with its homolog from molluscum contagiosum virus, MC021L [28]. The resulting virus, vMC021L-HA, has a plaque phenotype significantly smaller than its counterpart, vF13L-HA, but slightly larger than a full F13L deletion (vΔF13L) [28]. High and low MOI growth curves showed that vMC021L had similar growth kinetics compared to vF13L-HA but spreads slower in cell monolayers. Furthermore, similar levels of EV were produced by vMC021L-HA compared to vF13L-HA but the EV were less infectious [28]. Here, to further investigate the impact of MC021-HA expression on EV production, a novel flow virometry assay was used to reveal that MC021 increased the number of glycoproteins incorporated into released EV [29–42]. To expand the general understanding of the complex relationship between glycoprotein levels and viral infectivity, EV were sorted based on glycoprotein content utilizing fluorescence-activated virion sorting (FAVS). Sorted groups of EV containing different amounts of glycoproteins were further analyzed for infectivity, and produced a direct correlation of glycoprotein incorporation to the levels of infectious EV. Our findings were extended to demonstrate that manipulation of EV glycoprotein content was able to ameliorate lethality and pathology in a cutaneous challenge animal model. However, following cutaneous immunization, vMC021L-HA produced a similar titer of IMV neutralizing antibodies, and a similar profile of CD8^+^ T cell (T_CD8+_) responses to vF13L-HA. Therefore, manipulation of EV glycoprotein content around the optimal concentration could allow the design of bespoke viral vectors with a much-improved safety profile, without a compromise in immunogenicity in patients.

## Results

### Expression of MC021-HA results in EV with increased quantities of glycoprotein

**Fig 1:**
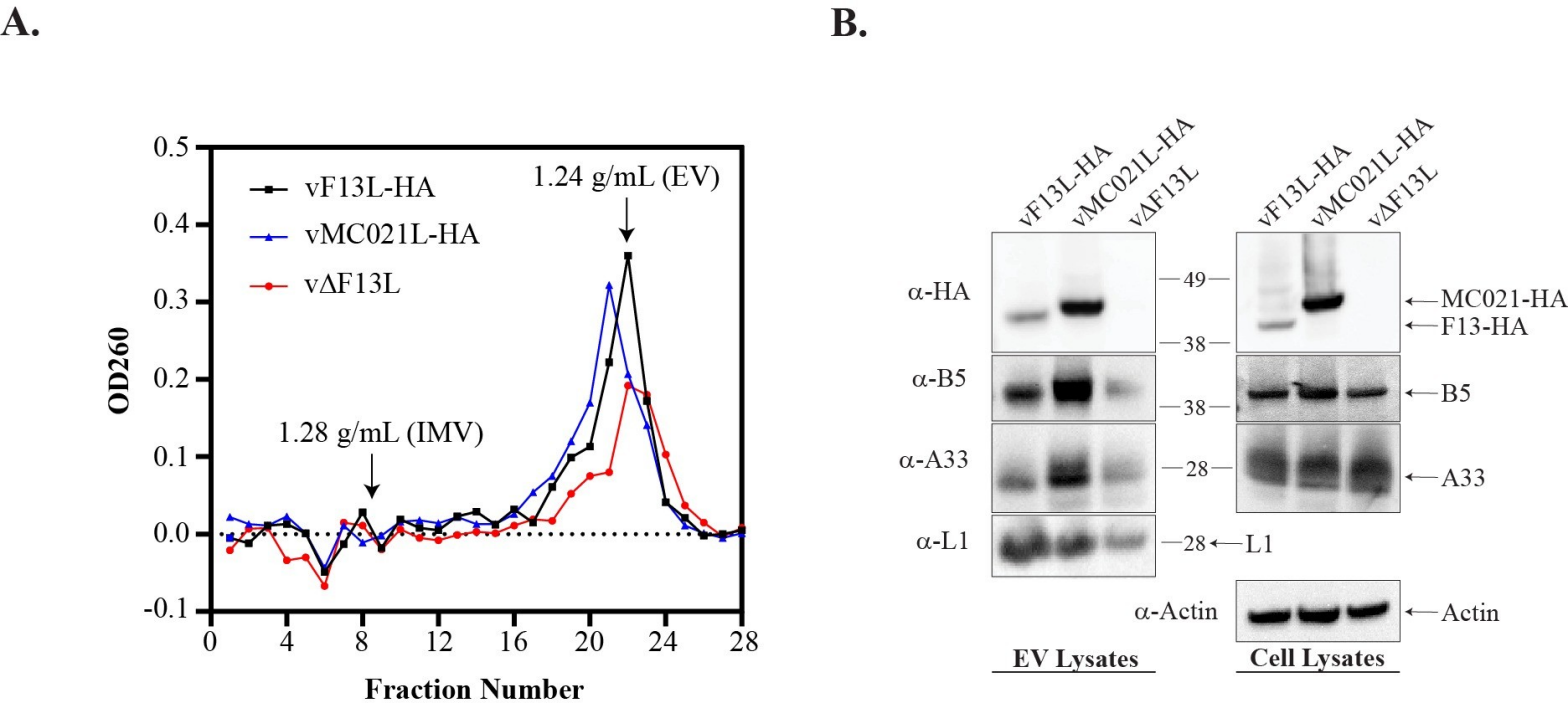
Glycoprotein content of EV membranes. (A) RK13 cells were infected with the indicated viruses at a MOI of 5. At 24 hpi, progeny virions released into the media (EV) were purified by CsCl density gradient centrifugation. Fractions from the gradient were collected dropwise from the bottom of the gradient and analyzed by OD260 and the concentration of CsCl determined by refractometry. (B) Fractions containing EV were collected (left) and infected cells were harvested (right) and analyzed by Western blot with rabbit anti-HA antiserum followed by HRP-conjugated donkey anti-rabbit antibody, rat anti-B5 MAb followed by Alexa Fluor 647-conjugated donkey anti-rat antibody, rabbit anti-A33 antiserum followed by HRP-conjugated donkey-anti rabbit antibody, rabbit anti-L1 antiserum followed by Alexa Fluor 647-conjugated donkey anti-rabbit antibody, and mouse anti-actin MAb followed by Alexa Fluor 647-conjugated donkey anti-mouse antibody. The masses in kilodaltons and positions of marker proteins are shown on the right of the EV lysate blot and to the left of the cell lysate blot.

A previous report from our lab determined that vMC021L-HA had similar replication kinetics as vF13L-HA, but produced a small plaque phenotype and EV that incorporated more MC021-HA compared to its homolog, F13-HA [28]. Glycoproteins, A33, A34, and B5, play multiple roles in EV target cell binding and outer membrane dissolution [22,23,43,44], and deletion or alteration of the incorporation of these glycoproteins results in the production of EV that are less infectious [16,17,19,20,22,23,43,44,45–47]. Considering that there are differences in the levels of F13/MC021, it seemed likely that vMC021-HA altered the glycoprotein content of EV, leading to their decreased infectivity. To determine if B5 and A33 were incorporated into the envelope of EV, EV released from cells infected with vF13L-HA/A4L-mCherry, vMC021L-HA/A4L-mCherry, and vΔF13L/A4L-mCherry (referred to as vF13L-HA, vMC021L-HA, and vΔF13L, respectively) were collected, purified, and separated from potential IMV in the media by CsCl gradient ultracentrifugation (Fig 1). A single peak of virions at 1.24 g/ml, representing EV, was present for each virus [48]. In contrast, little to no OD260 signal was detected at 1.28g/ml of CsCl where IMV are known to fractionate. Fractions at, and immediately adjacent to, 1.24 g/ml of CsCl were collected and the EV contained within were analyzed by Western blot (Fig 1B). EV produced by cells infected with vMC021L-HA incorporated more B5 and A33 compared to EV produced by vF13L-HA and vΔF13L (Fig 1B). Consistent with the previous finding, MC021-HA was incorporated to a greater extent than F13-HA [28]. The IMV protein L1 serves as a control to show the relative levels of virions analyzed. Western blot of infected cell lysates confirmed expression of F13-HA/MC021-HA, A33, and B5 (Fig 1B).

**Fig 2:**
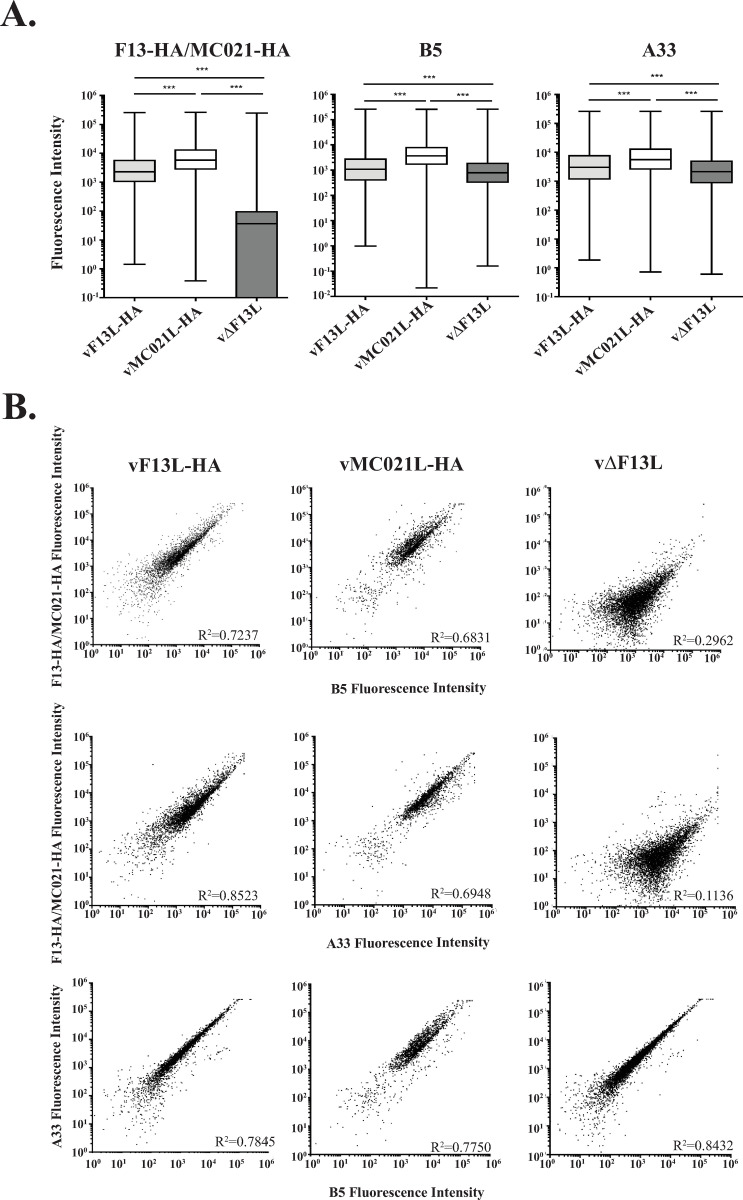
EV glycoprotein content analyzed by flow virometry. (A) RK13 cells were infected with the indicated viruses at a MOI of 5 and incubated at 37°C overnight. The next day, progeny virions released into the media (EV) were centrifuged through a sucrose cushion, fixed, and stained with rabbit anti-HA antiserum followed by Cy2-conjugated donkey anti-rabbit antibody, rat anti-B5 MAb followed by Dylight 405-conjugated donkey anti-rat antibody, and rabbit anti-A33 antiserum followed by Alexa Fluor 647-conjugated donkey anti-rabbit antibody. Shown are representative box and whisker plots for the incorporation of F13-HA/MC021-HA (left), B5 (middle), and A33 (right) in the EV membrane. ****, p<0.001 by Tukey’s multiple comparison test following one-way ANOVA. (B) Representative dot plots for the incorporation of B5 and F13-HA/MC021-HA (rows; top), A33 and F13-HA/MC021-HA (rows; middle), and A33 and B5 (rows; bottom) on viral surfaces for EV produced from vF13L-HA (columns; left), vMC021L-HA (columns; middle), and vΔF13L (columns right). All R^2^ values were significant (****, p<0.001).

The previous results suggest that an increased amount of F13-HA/MC021-HA, A33, and B5 was incorporated in the outer membrane of EV produced by vMC021L-HA, even though this virus exhibits a small plaque phenotype (Fig 1B) [28]. Therefore, a flow virometry assay was used to quantitatively examine glycoprotein incorporation for individual EV (Fig 2) [41,42]. Initial attempts to identify particles by flow virometry using forward and side scatter proved unreliable. All of the recombinant viruses express a core protein, A4, fused to mCherry. Thus, it should be possible to identify virions by mCherry fluorescence. To verify that virions could reliably be determined by their mCherry signal, purified EV were incubated with the fluorescent DNA stain Hoechst and imaged using confocal microscopy. In virion sized particles, the mCherry signal appeared relatively consistent and coincided with the DNA stain (S1 Fig) suggesting that mCherry fluorescence could be used to distinguish virions from debris. Using antibodies specific to A33, B5, and the HA epitope tag on either F13 or MC021, fixed VACV EV were fluorescently labeled. It should be noted that for comparison, both F13 and MC021 contain an identical HA epitope tag on their C-termini as it has been previously reported that the addition of the HA epitope tag to F13 has little-to-no effect on EV production and virus spread as compared to WR [28,49]. Using a flow cytometer, mCherry^+^ particles were analyzed for levels of F13-HA/MC021-HA (HA), A33, and B5. Consistent with Fig 1, EV produced by vMC021L-HA incorporated approximately twice as much B5 and thrice as much A33 compared to EV produced by vF13L-HA and vΔF13L EV, (Fig 2A). Similarly, more MC021-HA signal was detected in EV than F13L-HA while little-to-no signal was detected for EV produced by vΔF13L (Fig 2A). EV produced by vF13L-HA and vΔF13L EV contained similar amounts of B5 and A33 signal (vΔF13L EV incorporated approximately 1.16× the amount of A33 and 0.95× the amount of B5 compared to vF13L-HA), suggesting that the presence/absence of F13 did not affect the amount of B5 and A33 incorporated, confirming previous results [27].

To better understand the relationship between the detected proteins and their increased incorporation, EVs were plotted for their HA signals (x axis), i.e., F13 or MC021, against their A33 signals (y axis) (Fig 2B, top row), HA against B5 (Fig 2B, middle row), and A33 against B5 (Fig 2B, bottom row). Correlation values (R^2^) were calculated to determine if there was a linear relationship for protein incorporation. In general, there were high correlation values for B5:HA, B5:A33, and A33:HA for both vF13L-HA and vMC021L-HA indicating that for each increase in one of the proteins incorporated, there was an equal increase for the other two proteins. This linear relationship suggests that stoichiometric amounts of these proteins are incorporated during envelopment. In contrast, there was a low R^2^ value for the pairwise comparisons of HA signal to either B5 or A33 for vΔF13L (Fig 2B; right column, top and middle rows). Interestingly, the best correlation was seen for B5:A33 in virions produced by vΔF13L. Considering the correlation values were fairly similar between vF13L-HA and vMC021L-HA, it seems likely that MC021-HA increases protein incorporation overall but does not change the stoichiometric relationship of glycoprotein incorporation.

The increase in protein incorporation for vMC021L-HA EV could be the result of virion aggregation that would result in the simultaneous analysis of multiple virions (coincidental analysis), rather than a single virion. We theorized that the amount of the core protein should not vary, and therefore, the mCherry signal should be, somewhat consistent. To check this, the mCherry signal from the data shown in Fig 2 was plotted as histograms and the geometric mean of the signal intensities were calculated (S2 Fig). The plots are virtually superimposable with little variation in the geometric means demonstrating that even between the recombinant viruses, the mCherry fluorescent signal was consistent. The mCherry signal was plotted against HA, B5, and A33 to ascertain whether the detected increase in protein incorporation was the result of virion aggregation. For each virus, the mCherry signal had little to no significant correlation with B5, A33, or HA (Table 1) (S2 Fig) indicating that the level of these proteins was independent of the amount of core protein present. Therefore, virion aggregation does not account for the large differences in HA, B5, or A33 incorporation between the viruses and the increase in protein incorporation for EV produced by vMC021L-HA can be attributed to the expression of MC021-HA.

**Table 1:** Correlation of EV protein signal with mCherry fluorescence.

Virus	Protein	Pearson correlation coefficient (R^2^)
**vF13L-HA**	HA vs. mCherry	0.0001382
	B5 vs. mCherry	0.0001253
	A33 vs. mCherry	0.0001836
**vMC021L-HA**	HA vs. mCherry	0.0000431
	B5 vs. mCherry	0.0004555
	A33 vs. mCherry	0.0004181
**vΔF13L**	HA vs. mCherry	0.0000027
	B5 vs. mCherry	0.0000109
	A33 vs. mCherry	0.0000083

### B5-GFP can functionally replace B5 for EV production

**Fig 3:**
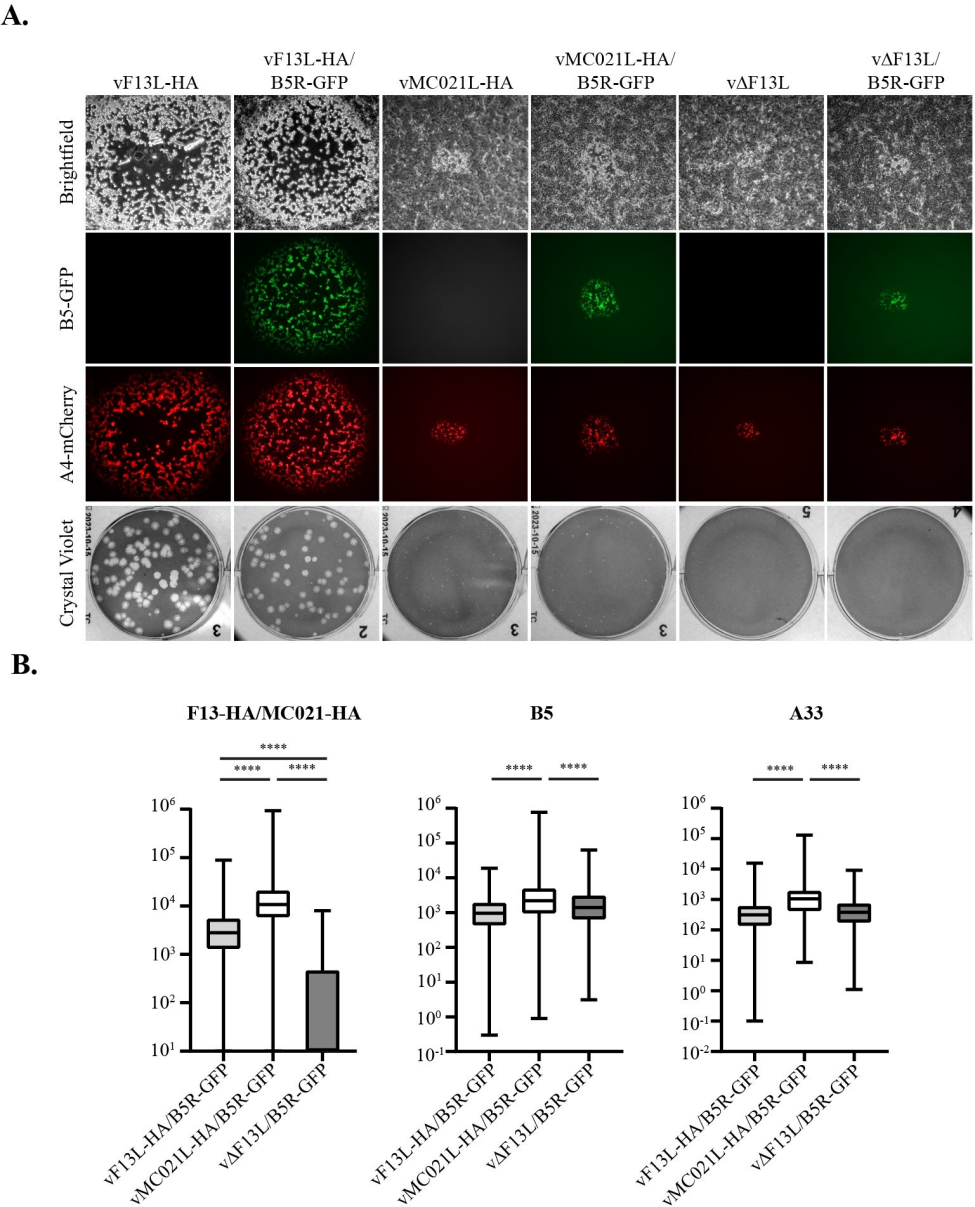
B5-GFP recombinant virus plaque phenotype and flow virometry. (A) Monolayers of BSC-40 cells were infected with the indicated viruses and incubated at 37°C overnight. After 2 h, the inoculum was removed, and cells were overlaid with semisolid medium. After 3 days, cell monolayers were imaged by fluorescence microscopy and then subsequently stained with crystal violet and imaged. (B) RK13 cells were infected with the indicated viruses at a MOI of 5 and incubated at 37°C overnight. The next day, EV were centrifuged through a sucrose cushion, fixed, and stained with rabbit anti-HA antiserum followed by Cy5-conjugated donkey anti-rabbit antibody, rat anti-B5 MAb followed by Dylight 405-conjugated donkey anti-rat antibody, and rabbit anti-A33 antiserum followed by Alexa Fluor 750-conjugated goat anti-rabbit antibody. Shown are representative box and whisker plots for the incorporation of F13-HA/MC021-HA (left), B5 (middle), A33 (middle), in the EV membrane. ****, p<0.001 by Tukey’s multiple comparison test following one-way ANOVA.

An abundance of previous evidence has shown that a decrease in A33, A34, and/or B5 incorporation correlates with a decrease in the infectivity of EV and a small plaque phenotype [14,16–18,20,23,26,43–46,50–59]. However, this is the first time an increase in glycoprotein incorporation has correlated to a defect in EV infectivity. These results suggest that there is an optimal glycoprotein concentration required for optimal EV infectivity. To test this hypothesis, the B5R gene in the three recombinant viruses was replaced with B5R-GFP [60,61] (now termed vF13L-HA/B5R-GFP, vMC021L-HA/B5R-GFP, and vΔF13L/B5R-GFP) to monitor the levels of the glycoprotein B5 in released EV without the use of fixation and antibodies. Previous work has demonstrated that the addition of GFP to B5 can functionally replace B5 during infection [51,60,61] and B5-GFP has proven to be a useful tool for studying IEV egress in living cells [62–65]. To verify that B5-GFP could functionally replace B5 in the context of vMC021L-HA infection, the plaque phenotypes of the A4L-mCherry parental viruses were compared to the new recombinants that express B5-GFP in place of B5 (Fig 3A). Comparison of the plaque phenotypes of these viruses revealed no discernable difference between vF13L-HA and vF13L-HA/B5R-GFP, vMC021L-HA and vMC021L-HA/B5R-GFP, and vΔF13L and vΔF13L/B5R-GFP, suggesting that B5-GFP can functionally replace B5 during infection in the context of this study.

### The addition of GFP to B5 does not impact the incorporation profile of HA, A33, and B5

It is possible that the expression of MC021-HA might impact the incorporation of B5-GFP relative to B5. To determine whether the incorporation profile of the A4L-mCherry/B5R-GFP recombinants looked similar to the incorporation profile of the A4L-mCherry parental viruses (Fig 2), EV produced by the new recombinant viruses were analyzed by flow virometry. Analysis of the data shows that an incorporation profile similar to the A4-mCherry parental viruses was observed for the B5-GFP viruses (Fig 3B) with vMC021L-HA/B5R-GFP EV incorporating approximately 2-3-fold more HA, A33, and B5 compared to vF13L-HA/B5R-GFP. Furthermore, correlation plots were generated where B5 signal (x axis) was plotted against A33 signal (y axis), HA against B5, and A33 against HA (S1 Table). Correlation values (R^2^) were calculated and were found to be similar to our previous results with the parental viruses (Fig 2B). Altogether, these results indicated that the B5-GFP viruses resembled the parental viruses in both functionality and pattern of glycoprotein incorporation. It should be pointed out that GFP fluorescence was poorly detected in these fixed samples necessitating the use of an antibody to detect B5-GFP incorporation. This was likely due to the denaturation of GFP by the ethanol fixation protocol used [66,67].

### EV containing an intermediate level of B5-GP have a higher specific infectivity compared to EV with either higher or lower B5-GFP content

**Fig 4:**
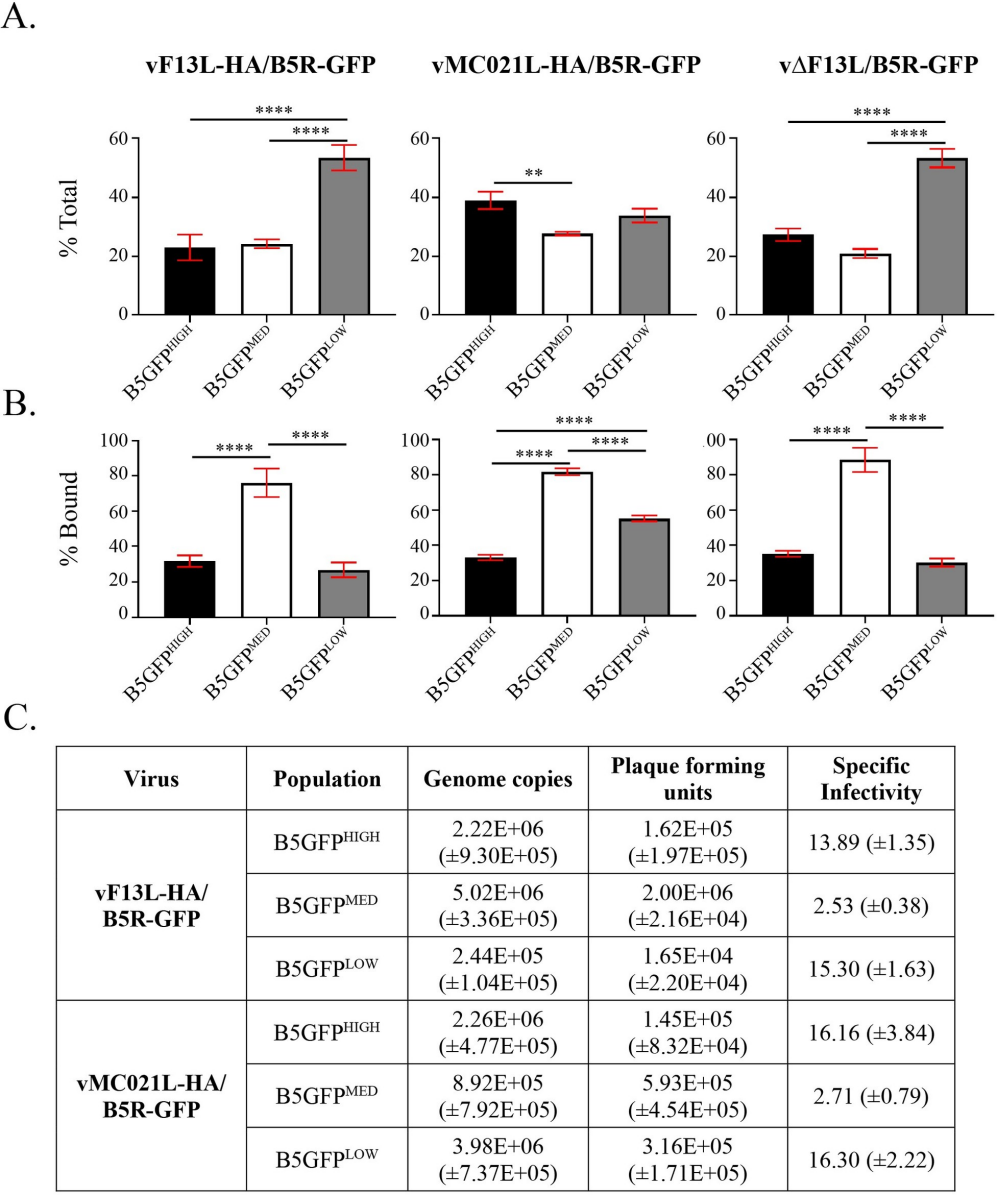
Fluorescence activated virion sorting and infectivity assays. RK13 cells were infected with the indicated viruses at a MOI of 5 and incubated at 37°C overnight. The next day, progeny virions released into the media (EV) were centrifuged through a sucrose cushion, and analyzed by fluorescence-activated virion sorting. EV virions were sorted into 3 pools based on GFP fluorescence intensity (B5GFP^HIGH^, B5GFP^MED^, and B5GFP^LOW^) and subsequently analyzed by qPCR for (A) total genome copies and (B) cell binding. (C) Specific infectivity was calculated by comparing total genome copies (by qPCR) and plaque forming units (by plaque assay). **, p<0.01 and ****, p<0.001 by Tukey’s multiple comparison test following one-way ANOVA.

After verifying that the B5-GFP viruses recapitulate the plaque phenotype and incorporation profile of the parental A4-mCherry viruses (Fig 3), a fluorescence activated virion sorting (FAVS) experiment was conducted using unfixed EV. As above, EV were identified by gating on mCherry^+^ events and then sorted based on high, medium, or low GFP fluorescence intensity (termed B5GFP^HIGH^, B5GFP^MED^, and B5GFP^LOW^, respectively; S3 Fig). Although sorting was conducted according to their B5-GFP content, based on the data in Fig 2B, the levels of A33 and F13/MC021 should correlate with the level of B5-GFP. Following EV sorting, the infectious properties of these three groups were investigated (Fig 4).

Considering that vMC021L-HA/B5R-GFP EV incorporate more B5 compared to both vF13L-HA/B5R-GFP and vΔF13L/B5R-GFP EV, it was expected that a larger percentage of the total amount of vMC021L-HA/B5R-GFP EV would fall within B5GFP^HIGH^. After sorting, virions in each group were concentrated and subsequently quantified using a sensitive qPCR assay in order to calculate the percentage of total virions that comprised B5GFP^HIGH^, B5GFP^MED^, and B5GFP^LOW^. For both vF13L-HA/B5R-GFP and vΔF13L/B5R-GFP, approximately 50% of the EV were either B5GFP^HIGH^ or B5GFP^MED^ and the other 50% was B5GFP^LOW^. However, as expected, for vMC021L-HA/B5R-GFP, the proportion of EV that were B5GFP^LOW^ shifted to B5GFP^HIGH^ and B5GFP^MED^, whereas approximately 70% of the EV were either B5GFP^HIGH^ or B5GFP^MED^ and only 30% was B5GFP^LOW^.

Next, EV from B5GFP^HIGH^, B5GFP^MED^, and B5GFP^LOW^ were tested for cell binding using a sensitive qPCR-based binding assay that measures genome copies [27,28,43,68] (Fig 4B). For vF13L-HA/B5R-GFP, B5GFP^MED^ EV exhibited the highest binding efficiency of approximately 76%, whereas B5GFP^HIGH^ and B5GFP^LOW^ EV displayed a significant reduction in binding efficiency compared to B5RGFP^MED^ of approximately 45–50%. A similar pattern was observed for both vΔF13L/B5R-GFP EV and vMC021L-HA/B5R-GFP EV, although in the latter case, B5GFP^LOW^ EV exhibited a significant reduction of approximately 25% in binding efficiency compared to B5RGFP^MED^. These results suggest that irrespective of the recombinant virus, EV containing an intermediate amount of B5 were better at binding cells than EV that have larger or smaller amounts of the glycoprotein.

Specific infectivity was calculated for B5GFP^HIGH^, B5GFP^MED^, and B5GFP^LOW^ EV produced by vF13L-HA/B5R-GFP and vMC021L-HA/B5R-GFP by comparing the total number of genome copies to plaque forming units (PFUs), (Fig 4C). Whereas 1 out of every 2.53 genome copies resulted in a plaque for B5GFP^MED^ EV for vF13L-HA/B5R-GFP, this number was elevated almost 5-fold to 1 out of every 13.89 and 15.30 for B5GFP^LOW^ and B5GFP^HIGH^ EV, respectively. Similarly, for vMC021L-HA/B5R-GFP, compared to B5GFP^MED^ EV, where 1 out of every 2.71 genome copies resulted in a plaque, B5GFP^LOW^ and B5GFP^HIGH^ EV exhibited an approximate 6-fold increase in specific infectivity, whereas 1 out of every 16.16 and 16.20 genome copies resulted in a plaque, respectively. Unfortunately, vΔF13L/B5R-GFP produced too few EV to sort and calculate a specific infectivity value [26]. Importantly, the specific infectivity data correlated with the binding data. Altogether, this data shows that there is a direct relationship between EV glycoprotein incorporation and infectivity, and furthermore, that an optimal or “just right” amount of glycoprotein must be incorporated to produce highly infectious EV, irrespective of the recombinant virus used.

### vMC021L-HA is attenuated, but retains immunogenicity, in vivo

**Fig 5:**
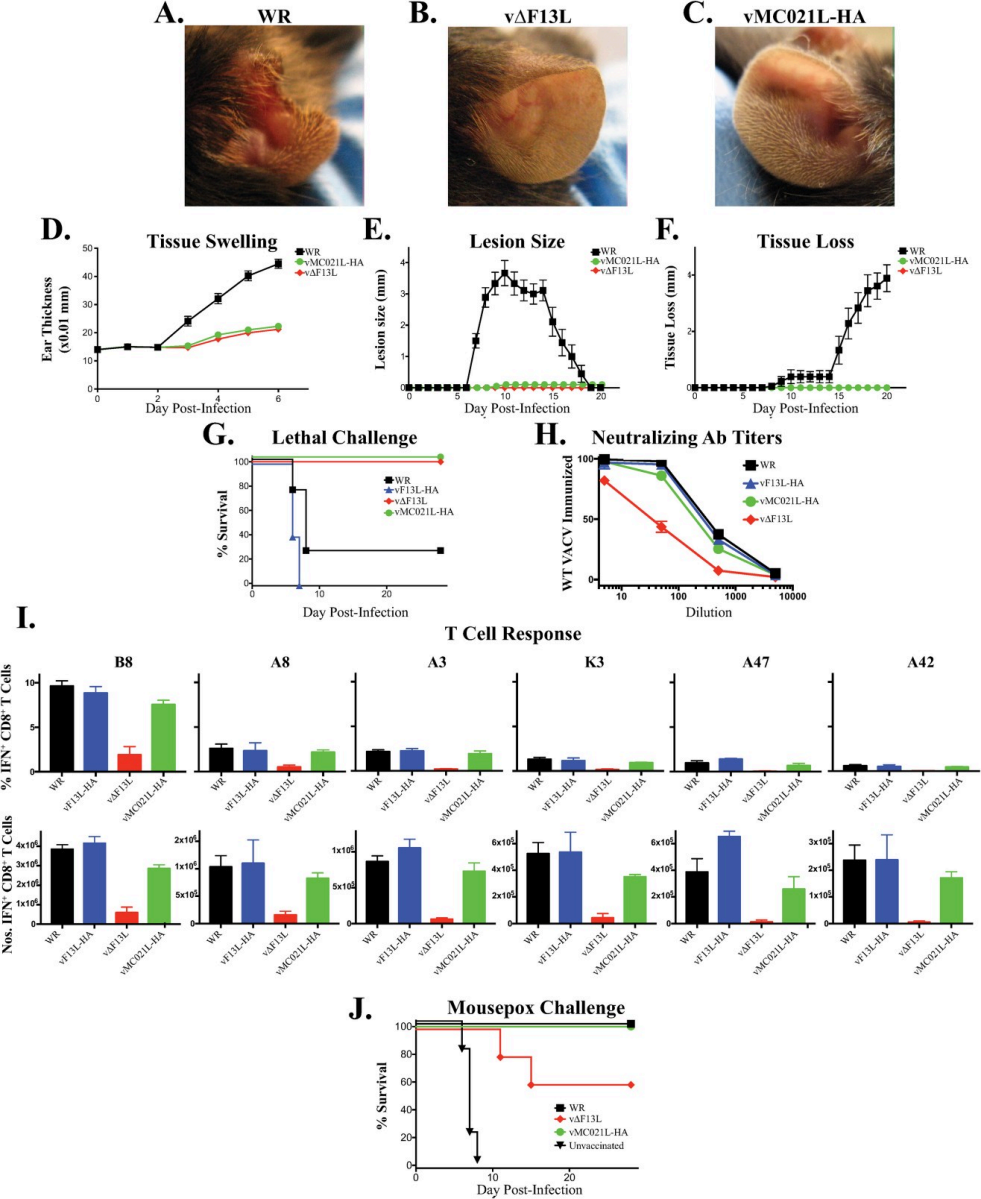
Pathogenesis and Immunity. (A-F) Wild-type C57Bl/6 mice were infected with 10,000 pfu of the indicated viruses in a single ear pinna and tissue pathology visualized (A-C), and tissue swelling (D), development of a lesion at the site of infection (E) and tissue loss at the site of infection (F) was measured. Graphs showed pooled data from two experiments (n = 10). (G) STAT1^-/-^ mice (n ≥3) were infected intradermally with 10,000 pfu of the indicated viruses in each ear pinna and survival was monitored for 28 days post-infection. Graph is representative of pooled data from two experiments (n = 7–10). (H) Wild-type C57Bl/6 mice were immunized with 10,000 pfu of the indicated viruses in a single ear pinna, serum harvested 38 days later and the ability of serum dilutions to inhibit infection with WR-GFP assayed by flow cytometry. (I) Wild-type C57Bl/6 mice were immunized with 10,000 pfu of the indicated viruses in a single ear pinna, and the splenic CD8+ T cell responses to immunodominant B8, A8, A3, K3, A47 and A42 determinants assayed by conventional intracellular cytokine secretion assay. (J) Wild-type Balb/c mice were immunized with 10,000 pfu of the indicated viruses in a single ear pinna, then 35 days later challenged by footpad infection with 3000 pfu of the virulent mouse pathogen, ECTV. Survival of mice is shown from two combined experiments (n = 10).

Previously it was reported that vMC021L-HA produced as much EV as vF13L-HA, but exhibits a small plaque phenotype, albeit slightly larger than vΔF13L [28] (Fig 3A). Moreover, vMC021L-HA EV have increased amounts of glycoproteins A33 and B5, both of which have been shown to be the targets of neutralizing antibodies [3–8]. Given these results, it was hypothesized that vMC021L-HA would be attenuated *in vivo*, but the slight increase in spread seen by vMC021L-HA, compared to vΔF13L, coupled with the release of viral antigens may nonetheless allow generation of protective immune responses by this virus. To test this hypothesis, we infected wild-type mice intradermally with the IMV form of the virus, a route that mimics both the natural route of infection and the route of immunization with vaccinia in the smallpox virus eradication program [69–72]. 14 days after intradermal infection, tissue pathology could be easily identified in mice infected with VACV WR (Western Reserve) (Fig 5A), but no pathology was readily observable after infection with either vΔF13L (Fig 5B) or vMC021L-HA (Fig 5C). To quantify these observations, we measured the characteristic swelling (Fig 5D), lesion development (Fig 5E) and tissue loss (Fig 5F) normally associated with dermal VACV infection [73]. Although infection with either vΔF13L or vMC021L-HA did trigger some local tissue swelling, this was much reduced compared to infection with VACV WR, and we did not observe development of a lesion, or subsequent tissue loss after infection with either vΔF13L or vMC021L-HA. STAT1^-/-^ mice are particularly sensitive to orthopoxvirus infection [74], and we have found that this sensitivity extends to cutaneous infection with limited doses of virus (Fig 5G). Following infection with vF13L-HA, all STAT1^-/-^ mice died by 8 days post-infection. However, all mice infected with either vMC021L-HA or vΔF13L-HA survived to the end of the experiment at 28 days post-infection. Therefore, vMC021L-HA and vΔF13L-HA are attenuated *in vivo* and that the increase in EV glycoprotein exhibited by vMC021L-HA contributes to this attenuation.

To assess whether the decrease in EV infectivity caused a commensurate reduction in immunogenicity *in vivo*, we infected wild-type mice intradermally with VACV WR, vF13L-HA, vΔF13L or vMC021L-HA and harvested serum 38d after infection. We then examined the ability of dilutions of serum from each cohort of infected animals to block VACV-GFP *in vitro* using an assay that predominantly measures IMV neutralization [75]. Immunization with either VACV WR or vF13L-HA produced serum antibodies that blocked 50% of infection at a dilution of ~1:250, whereas immunization with vΔF13L only produced serum antibodies that blocked 50% of infection at a dilution of ~1:30 (Fig 5H). However, we found that immunization with vMC021L-HA produced serum antibodies that blocked 50% of infection at levels only just below those immunized with VACV WR, at a dilution of ~1:200 (Fig 5H). A potent T_CD8+_ response, that targets multiple epitopes, including the B8, A8, A3, K3, A47 and A42 epitopes in mice on an H2^b^ background, is also induced by VACV WR 8 days after infection (Fig 5I) [76]. A similar T cell response is observed after immunization with vF13L-HA, but immunization with vΔF13L induced a much-reduced response to all the determinants examined and was undetectable above background to the A47 and A42 epitopes (Fig 5I). With vMC021L-HA, we observed a T cell response to all determinants that was markedly above that induced following immunization with the vΔF13L, and often approached that observed after immunization with VACV WR or vF13L-HA. Therefore, the immunogenicity of vMC021L-HA appears intact, despite both a marked diminution in spread *in vitro* and induction of pathology *in vivo*. To assess the functionality of our correlates of protective immunity, we immunized susceptible Balb/c mice with VACV WR, vΔF13L or vMC021L-HA and challenged with the virulent mouse poxvirus ectromelia (ECTV) 35 days later. Mice that had not been immunized all died within 8 days of ECTV infection, while 40% of those immunized with vΔF13 died between day 10 and 20 post-infection, a time point that likely indicates a failure of the adaptive immune system to contain the virulent infection. However, mice immunized with either VACV WR or vMC021L-HA all survived the challenge, indicating the induction of functional protective immunity by vMC021L-HA.

## Discussion

VACV was a vital component of the largest and most successful vaccination program in history, producing protective immunity against smallpox, and currently is the basis for many viral vaccines, oncolytics, and gene therapy vectors. However, there are numerous complications arising from the use of VACV in patients, the majority of which stems from uncontrolled replication and spread of the virus from the original site of administration. Therefore, the design of non-replicating and/or non-spreading VACV-based vectors may appear to be a favorable approach. However, the immunogenicity of VACV-based vectors is closely correlated with their ability to spread *in vivo* [76], and nonreplicating vectors, such as the Modified Virus Ankara (MVA), display a marked diminution in the ability to induce protective immune responses in mammals necessitating multiple doses [76]. Therefore, it is crucial to gain a greater understanding of the mechanisms that governs virus replication, assembly and spread, in order to design viral vectors in which the factors governing spread can be identified and separated from those governing immunogenicity *in vivo*.

To this end, we investigated the role of the EV protein F13, which plays a critical role in the production of infectious EV, as its deletion results in a severe reduction in plaque size. Indeed, F13 has previously been shown to have two roles, first the production of wrapped virus (IEV and EV) [26], and a second role in EV infectivity via cell entry [27]. The current study suggests that F13 controls the amount of glycoprotein incorporated into the outer envelope of EV during intracellular envelopment. At this time, it is unclear how F13 limits glycoprotein incorporation while the molluscum contagiosum virus homolog facilitates greater incorporation. One possibility may arise from the increased interaction seen between MC021 and A33 [28]. As A33, A34 and B5 are known to form a complex [44] it is possible that the increased A33-MC021 interaction allows for greater incorporation into the envelope. Concurrently, there is an increase in the amount of MC021 found in the envelope (Figs 1B and 2A). The addition of GFP to the cytoplasmic tail of B5 abrogates its interaction with F13 and MC021 [28]. The effect of this abrogation is only apparent when A33 is absent [23] suggesting that A33 can mediate the incorporation of B5 into the envelope of released virions and the increase in B5 seen in the presence of MC021 is through an increased interaction with A33. The strong correlation between the amount of A33, B5, and F13/MC021 found in each virion indicates a stoichiometric relationship between their incorporation, further suggesting that at least these three proteins are incorporated as a complex.

Even though F13L-HA and MC021L-HA were both expressed from an identical F13L promoter, a greater amount of MC021-HA was detected in lysates from infected cells compared to F13-HA (Fig 1B). The increased level of MC021 could also account for the increased envelope incorporation by facilitating interactions between EV proteins and proteins on the surface of IMV. It is highly likely that at least one of the four critical EV proteins (A33, A34, B5, and F13) if not all, serves as a matrix-like protein or protein complex to facilitate envelopment of IMV to form EV. Thus, an increase in formation of a matrix-like complex should result in increased interactions between EV and IMV proteins, accounting for their increased incorporation into released virions. Further work is required to better understand the molecular aspects of intracellular envelopment and the functions of A33, A34, B5 and F13 in this process.

Previous reports have used flow virometry to study herpes simplex virus 1 (HSV-1), Junin viruses, HIV, Nipah, dengue, human cytomegalovirus (HCMV), and T4 and lambda phages [29–42]. Some of these studies reported sorting virions based on either size, surface protein content or conformation, and/or genome content and testing these sorted populations for infectivity [34,41,42]. Flow virometric analysis of VACV virus stocks has also been reported for the evaluation of vaccine preparations [77]. However, here we utilize this technique to analyze the relative protein content of VACV EV (Figs 2 and 3) to directly correlate glycoprotein content to cell binding and subsequent infectivity (Fig 4). Surprisingly, flow virometric analysis revealed a large range of protein incorporation per virion for all three of the recombinant viruses analyzed, suggesting a stochastic process for envelope composition (Figs 2 and 3). Using western blots with purified extraviral portions of EEV proteins expressed in either insect cells (B5 and A33) or bacteria (A34), the amount (ug) of these three glycoproteins /mg of EEV was calculated [6]. Using this method, A34 and B5 were reported to have approximately equal abundance (40 and 30 ug/mg EEV) whereas much less, if any, (<5ug/mg) A33 was detected. Our results here neither confirm nor refute these assertions as we did not determine absolute glycoprotein concentration per virion making it difficult to directly compare these previous results to those reported here. If A33, B5, and A34 are incorporated as a complex, then it is possible that the complex does not contain equal molar ratios of each glycoprotein.

Our analysis suggests that it is the increase in glycoprotein content that leads to a decrease in cell binding and an overall reduction in the infectivity of vMC021L-HA. It is generally accepted that in multivalent systems, such as virion glycoproteins interacting with cellular receptors, an increase in the number of ligands (glycoproteins) on the surface of the particle should lead to an increase in binding affinity [78–80]. While it is easy to conceptualize how a decrease in glycoprotein content could lead to a decrease in cell binding, we were surprised to find that an overall increase in glycoprotein density on the virion surface was equally detrimental to cell binding. This suggests a possible regulatory mechanism to control cell binding that is not fully understood.

F13 appears to limit the relative amounts of EV glycoproteins incorporated in the outer EV membrane, and that there is an optimal concentration of glycoproteins required for infectivity. An intriguing side effect of changing the concentration of EV glycoproteins in the outer membrane is that virions are produced from infected cells in normal quantities and display enhanced levels of EV proteins, which potentially could be targets for a neutralizing antibody response. The result is a recombinant VACV that has reduced spread and does not cause any discernable pathology upon immunization, but which produces neutralizing antibodies at levels approaching those induced upon infection with wild-type VACV. Although not directly assessed in this study, it is possible that compared to vΔF13L, vMCO21L-HA induced an increased antibody response against EV proteins that was similar to the response induced by vF13L-HA. Surprisingly, vMC021L-HA induced strong T_CD8+_ responses, a hallmark of VACV that spreads effectively [76]. However, immunization with inactivated VACV virions can induce an effective T_CD8+_ response [81], so it is possible that the production of EV by cells infected at the site of immunization produces sufficient virus particles to reproduce, or even exceed this immunogenicity. In addition, when one considers the increased immune response generated by vMC021L-HA compared to vΔF13L, it seems likely that the slight increase in spread (Fig 3) may have been enough to elicit a robust immune response. Furthermore, these data raise the very real possibility that careful manipulation of EV glycoprotein content can produce, effective immunogenic viral vectors that are attenuated such that they do not display the side effects of traditional VACV vectors, but which display a marked enhancement in immunogenicity relative to non-replicating vectors. Currently licensed vaccines against smallpox include two attenuated strains of vaccinia viruses LC16m8 and MVA. LC16m8 was derived from the Lister vaccine strain and been licensed since 1975 in Japan [82]. LC16m8 has been reported to be temperature-sensitive and have a truncation in the EV glycoprotein B5 [83]. Although attenuated, LC16m8 is replication competent and has been shown to induce immune responses similar to both Dryvax and Lister [83,84]. MVA is a third-generation smallpox vaccine that is licensed in Europe, Canada, and the US for immunization against smallpox and monkeypox [85]. MVA was attenuated by multiple passages of the vaccinia virus Ankara strain in chicken embryo fibroblasts [86]. The resulting virus has large deletions in its genome and is missing multiple immunomodulatory genes resulting in severe attenuation and the inability to fully replicate in mammalian cells [86,87]. The virus has a very good safety profile and is considered safe even in immunocompromised individuals but requires two doses. Both MVA and LC16m8 demonstrate that attenuated vaccinia viruses can be used as effective orthopoxvirus vaccines. Future studies will require the marriage of traditional virology studies of replication, assembly and spread, with the *in vivo* study of mechanisms of pathogenesis, the study of multiple intricate mechanisms of immune induction, and the study of the correlates and longevity of protection in challenge models.

## Materials and methods

### Ethics statement

The Research Subjects Review Board of the University of Rochester Medical Center approved this research. All animals were maintained in the specific-pathogen-free facility of the Hershey Medical Center and treated in accordance with the National Institutes of Health and AAALAC International regulations. All animal experiments and procedures were approved by the Penn State Hershey Institutional Animal Care and Use Committee that follows the Office of Laboratory Animal Welfare PHS Policy on Humane Care and Use of Laboratory Animals, 2015.

### Cells

BSC-40 cells were obtained from ATCC and maintained in Dulbecco’s Modified Eagle’s Medium (DMEM) supplemented with 10% fetal bovine serum (FBS). RK13 cells were obtained from ATCC and maintained in Earle’s Minimum Essential Medium (EMEM) supplemented with 10% FBS.

### Viruses and infections

vF13L-HA and vΔF13L were generously provided by Bernard Moss (National Institutes of Health, Bethesda), and their generation has been described previously [26,88–90]. vMC021L-HA has been described previously [28]. vF13L-HA/A4L-mCherry and vΔF13L/A4-mCherry have been described previously [27]. vMC021L-HA/A4L-mCherry was generated by infecting HeLa cells with vMC021L-HA followed by transfection with a plasmid encoding the red fluorescent protein, mCherry, fused to the N terminus of A4 with 500-bp flanking homology, and screened for by the production of red plaques. The MC021L/F13L locus was sequenced in all of the viruses used in this study to verify that it is correct. vF13L-HA/B5R-GFP/A4L-mCherry, vMC021L-HA/B5R-GFP/A4L-mCherry, and vΔF13L/B5R-GFP/A4L-mCherry were generated by infecting HeLa cells with vF13L-HA/A4L-mCherry, vMC021L-HA/A4L-mCherry, and vΔF13L/A4L-mCherry, respectively, followed by transfection with pB5R-GFP [60,61] and screened by the production of green and red plaques. WR-GFP was previously described [91].

### Mice

C57BL/6 or Balb/c mice were purchased from Charles River Laboratories or Jackson Laboratories. Breeding pairs of STAT1^+/-^ mice were purchased from Jackson Laboratories and bred in the specific-pathogen-free animal facility at the Penn State Hershey College of Medicine. For intradermal (i.d.) VACV infections, mice aged 7–10 weeks were anesthetized with ketamine/xylazine and injected with 10^4^ PFU of VACV in <10 μL in each ear pinna. For footpad ECTV infections, mice were injected with 3000 PFU ECTV Moscow in the right footpad. During lethal challenge of STAT1^-/-^ with VACV, or of Balb/c mice with ECTV, mice were monitored for death twice daily for 28 days following infection. To monitor pathogenesis in the ears, ear thickness was measured using a 0.0001 in. micrometer (Mitutoyo, Aurora, IL). Lesion progression and subsequent tissue loss were measured daily.

### Plaque assays and virus quantification

Plaque assays were conducted as previously described [28]. Plaques were imaged after 3 days using a Leica DMIRB inverted fluorescence microscope with a cooled charge-coupled device (Cooke) controlled by Image-Pro Plus software (Media Cybernetics). Images were compiled and minimally processed using Photoshop (Adobe). After microscopic imaging of plaques, monolayers were stained with crystal violet. Viral genomes were quantified by qPCR as described previously [68].

### Analysis of EV

EV purification by CsCl gradient was conducted as described previously [28]. Briefly, EV from a clarified supernatant of infected cells was initially pelleted through a 36% sucrose cushion. The resulting virus pellet was resuspended in 10mM Tris (pH 9.0) and a portion was used in a dot blot with an anti-L1 antibody to determine the relative levels of virions in each pellet. The rest of the pellet was layered on to a CsCl step-gradient (1.3 g/ml, 1.25g/ml, and 1.2 g/ml CsCl) and centrifuged at 77,175 × g for 95 min. Fractions (1ml) were collected from the bottom for analysis. For Western blotting, virus containing fractions were diluted in 10 mM Tris (pH 9.0) and centrifuged at 100,000 × g for 1 h. Pellets were diluted in protein gel sample buffer, and equilibrated volumes, as determined by the post-sucrose cushion anti-L1 dot blot, were analyzed by Western blotting as described previously [43]. The following antibodies were used: rabbit anti-HA antiserum (Sigma), rat anti-B5 MAb [12], rabbit anti-A33 antiserum (NR-628; BEI Resources), mouse anti-actin MAb (Sigma), and rabbit anti-L1 antiserum (NR-631; BEI-Resources). HRP- and Alexa Fluor 647-conjugated donkey anti-rat and anti-mouse antibodies were purchased from Jackson ImmunoResearch Laboratories. HRP was detected using chemiluminescent reagents (Pierce) following the manufacturer’s instructions. The fluorescent and chemiluminescent signal was captured using a Kodak Image Station 4000mm Pro (Carestream Health Inc.).

### Flow virometry

RK13 cells were infected with the indicated viruses at a MOI of 5 at 37°C. The following day, cell culture supernatants were collected and clarified by low-speed centrifugation at 913 × g for 10 min. Clarified supernatants were mixed with filtered 100% ethanol to a final concentration of 70% ethanol and incubated overnight at room temperature. The next day the fixed virions were clarified by low-speed centrifugation at 913 × g for 10 min. Clarified supernatants were overlaid on a 36% sucrose cushion and centrifuged at 100,000 × g for 40 min to pellet EV. EV were resuspended in Tris-EDTA (TE) buffer containing primary antibodies, as described in the figure legends, and incubated for 3 hrs at 4°C. EV were then pelleted at 16,000 × g for 10 min, washed three times with TE buffer, and resuspended in TE buffer containing secondary antibodies, as described in the figure legends, and incubated at 4°C. 3 hrs later, EV were pelleted, washed, and resuspended in TE buffer as described above. Stained EV were analyzed with an LSRII-18 color BD Biosciences flow cytometer, using appropriate lasers and filters. Virions were distinguished from debris by gating for mCherry^+^ events (A4-mCherry) and mCherry^+^ events were gated for Cy2 (HA or B5-GFP), Alexa Fluor 647 (A33 or HA), Dylight 405 (B5), and Alexa Fluor 750 (A33) positive events, as described in the figure legends. The following antibodies were used: rabbit anti-HA antiserum (Sigma), rat anti-B5 MAb [12], and mouse anti-A33 MAb (NR-49231; BEI Resources). Alexa Fluor 647-conjugated donkey anti-mouse and anti-rabbit antibodies, Dylight 405-conjugated donkey anti-rat antibody, and Cy2-conjugated donkey anti-rabbit antibody were purchased from Jackson ImmunoResearch Laboratories. Alexa Fluor 750-conjugated goat anti-mouse antibody was purchased from Invitrogen. All data were analyzed using FACSDiva 8.0.1 (BD Biosciences) and FCS Express 7 (De Novo Software).

### Confocal microscopy

EV from cells infected with vF13-HA were purified from clarified supernatants as described above except the ethanol fixation step was omitted. Unfixed, purified EV were washed twice with 10mM Tris (pH 9.0) and resuspended 10mM Tris (pH 9.0) containing 10μg/ml Hoechst 34580 (AdipoGen). After 1h, EV were pelleted at 16,000 × g for 10 min, washed thrice with 10mM Tris (pH 9.0) and resuspended in 100 μl of 10mM Tris (pH 9.0). 30 μl of resuspended EV was spotted onto a #1.5 coverslip and incubated at room temperature for 1h. After which, coverslips were washed twice with 10mM Tris (pH 9.0) and mounted with ProLong Diamond (ThermoFisher). Mounted coverslips were imaged using a Nikon A1R HD laser scanning confocal microscope as a z-series. Collected z-stacks were deconvoluted using the NIS-Elements C software (Nikon) and maximum intensity projections were created using ImarisViewer software (Oxford Instruments).

### Fluorescence activated virion sorting

RK13 cells were infected with the indicated viruses at a MOI of 5 at 37°C. The following day, cell culture supernatants containing released EV were collected and clarified by low-speed centrifugation at 913 × g for 10 min, overlaid on a 36% sucrose cushion, and centrifuged at 100,000 × g for 40 min to pellet EV. EV were resuspended in TE buffer and sorted in a BSL-2 facility with a BD FACSAria II flow cytometer, using appropriate lasers and filters. Positive virions were first isolated by gating for mCherry^+^ events (A4-mCherry) through a 610/20 bandpass filter and sorted based on high, medium, and low GFP fluorescence emission through a 525/50 bandpass filter. Sorted EV were concentrated by centrifugation at 16,000 × g for 10 min and the resulting pellet was resuspended in TE buffer for qPCR, binding, and plaque assays.

### EV cell binding

A virus binding assay was performed and quantified by qPCR as described previously [27,28,43,44,68]. Briefly, RK13 cells were infected with the indicated viruses at a MOI of 5 and incubated at 37°C overnight. The next day, EV were centrifuged through a sucrose cushion, and analyzed by fluorescence-activated virion sorting into 3 pools based on GFP fluorescence intensity (B5GFP^HIGH^, B5GFP^MED^, and B5GFP^LOW^). Aliquots from each sorted virus pools were treated with DNase I before genomes were purified and analyzed by qPCR. Monolayers of BSC-40 cells were treated with PBS containing 0.6 mM EDTA for 15 min at 37°C to detach cells. Cells were counted and 10^5^ cells were suspended in the aliquot from the sorted EV samples and rotated at 4°C for 1 h. After, cells were pelleted, and viral DNA was quantified by qPCR.

### Neutralization assay

A flow cytometric assay for measuring neutralizing antibody titers has been previously described [75]. Briefly, wild-type C57Bl/6 mice were immunized with 10,000 pfu of the indicated viruses in a single ear pinna. Serum was harvested 38 days later, and serial dilutions of the serum were incubated with a stock of WR-GFP for an hour. The stock was generated from a lysate of cells infected with WR-GFP and therefore potentially contains both IMV and EV, but would predominantly be IMV. Subsequently, the ability of WR-GFP incubated with various dilutions of serum to infect cells was measured by incubating with HeLa cells at an MOI of 5, then allowing expression of GFP for 6 hs. Infected cells were fixed and analyzed for GFP expression using a BD Fortessa Flow cytometer.

### Intracellular cytokine staining assay

Single cell suspensions generated from spleen, lymphocytes were isolated by centrifugation over Lymphocyte Separation Medium (Cambrex) and then stimulated for 4 h with 1 μM of each VACV peptide prior to the addition of 10 μg/mL brefeldin A (BFA; Sigma). VACV-derived peptides B8, A8, A3, K3, A47 and A42 have been previously described [92]. Following peptide stimulation, cells were blocked in 2.4G2 supernatant containing 10% mouse normal mouse serum and then stained for CD8. Cells were fixed in 2% paraformaldehyde then permeabilized and stained for intracellular IFN-γ in 2.4G2 supernatant supplemented with 10% normal mouse serum and 0.5% saponin. Net frequencies and numbers of cytokine-positive T_CD8+_ were calculated by subtracting the unstimulated background response.

## Supporting information

S1 Fig Imaging of purified virions. RK13 cells were infected with vF13L-HA at a MOI of 5 for 24 h. Extracellular virions were purified as described, stained with Hoechst 34580, mounted in ProLong Diamond, and imaged using confocal microscopy. A representative field of view is shown. Red represents mCherry fluorescence and blue staining is Hoechst 34580. Pink represents the overlap of red and blue signal. Scale bar is 3 μm.
(TIF)

S2 Fig Flow virometry. (A) RK13 cells were infected with vF13L-HA (left panel) or indicated viruses (right panel) at a MOI of 5 for 24 h. After, extracellular virions were purified as described, fixed, and analyzed by flow cytometry for mCherry fluorescence intensity. (Left Panel) Percentages represent the percent of events in each box relative to the total number of events both inside and outside of the boxes. (note some events fall outside of the red boxes). (Right Panel) Histogram showing the number of events at each mCherry fluorescence intensity for the indicated viruses. The calculated geometric mean for each virus is shown in the table. (B) The mCherry^+^ population, denoting virions, was analyzed for glycoprotein incorporation. Representative dot blots for the incorporation of mCherry and F13-HA/MC021-HA (rows; top), mCherry and A33 (rows; middle), and mCherry and B5 (rows; bottom) on viral surfaces for EV produced from vF13L-HA (columns; left), vMC021L-HA (columns; middle), and vΔF13L (columns right).
(TIF)

S3 Fig Fluorescence activated virion sorting scheme. RK13 cells were infected with vF13L-HA/B5R-GFP at a MOI of 5 for 24 h. Extracellular virions were centrifuged through a sucrose cushion and analyzed by fluorescence-activated virion sorting. Virions were first gated on mCherry fluorescence intensity as in S1 Fig and mCherry^+^ events were subsequently sorted based on GFP fluorescence intensity (B5GFP^HIGH^, B5GFP^MED^, and B5GFP^LOW^). Y-axis is count normalized for the number of sorted events (n) for easier visualization, and n is denoted for each population.
(TIF)

S1 Table Correlations of detected signal for B5-GFP recombinant viruses. Data from Fig 3B was used to generate dot plots of the detected signal for each particle analyzed. The resulting plots were analyzed for linear correlation (R2) using Pearson correlation coefficient. ^a^ ****, p<0.001.
(DOCX)
