# Supplementary figures and images for: An increase in glycoprotein concentration on extracellular virions dramatically alters vaccinia virus infectivity and pathogenesis without impacting immunogenicity
Source: PLoS Pathog. 2021 Dec 28;17(12):e1010177. doi: 10.1371/journal.ppat.1010177 (PMC8746760; doi:10.1371/journal.ppat.1010177)

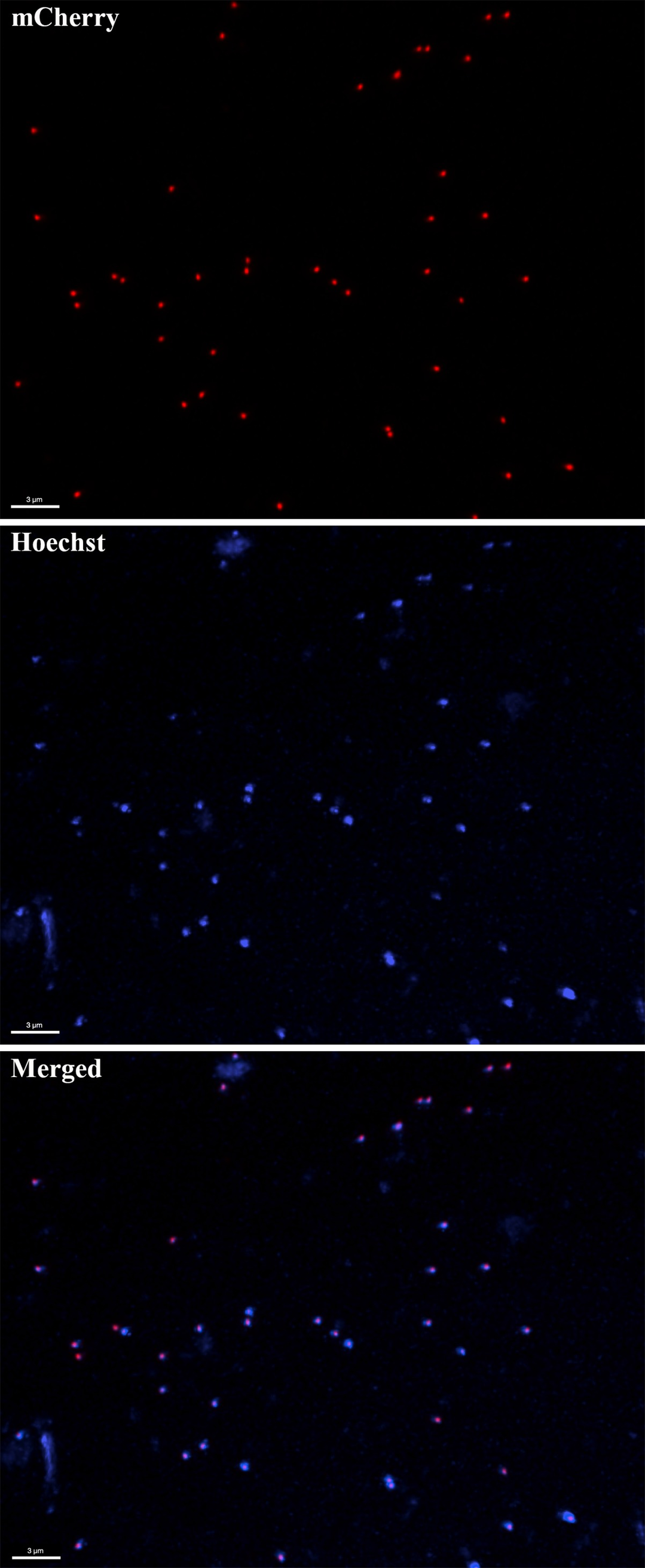

Supplement: S1 Fig — RK13 cells were infected with vF13L-HA at a MOI of 5 for 24 h. Extracellular virions were purified as described, stained with Hoechst 34580, mounted in ProLong Diamond, and imaged using confocal microscopy. A representative field of view is shown. Red represents mCherry fluorescence and blue staining is Hoechst 34580. Pink represents the overlap of red and blue signal. Scale bar is 3 μm. (TIF) [file ppat.1010177.s001.tif]

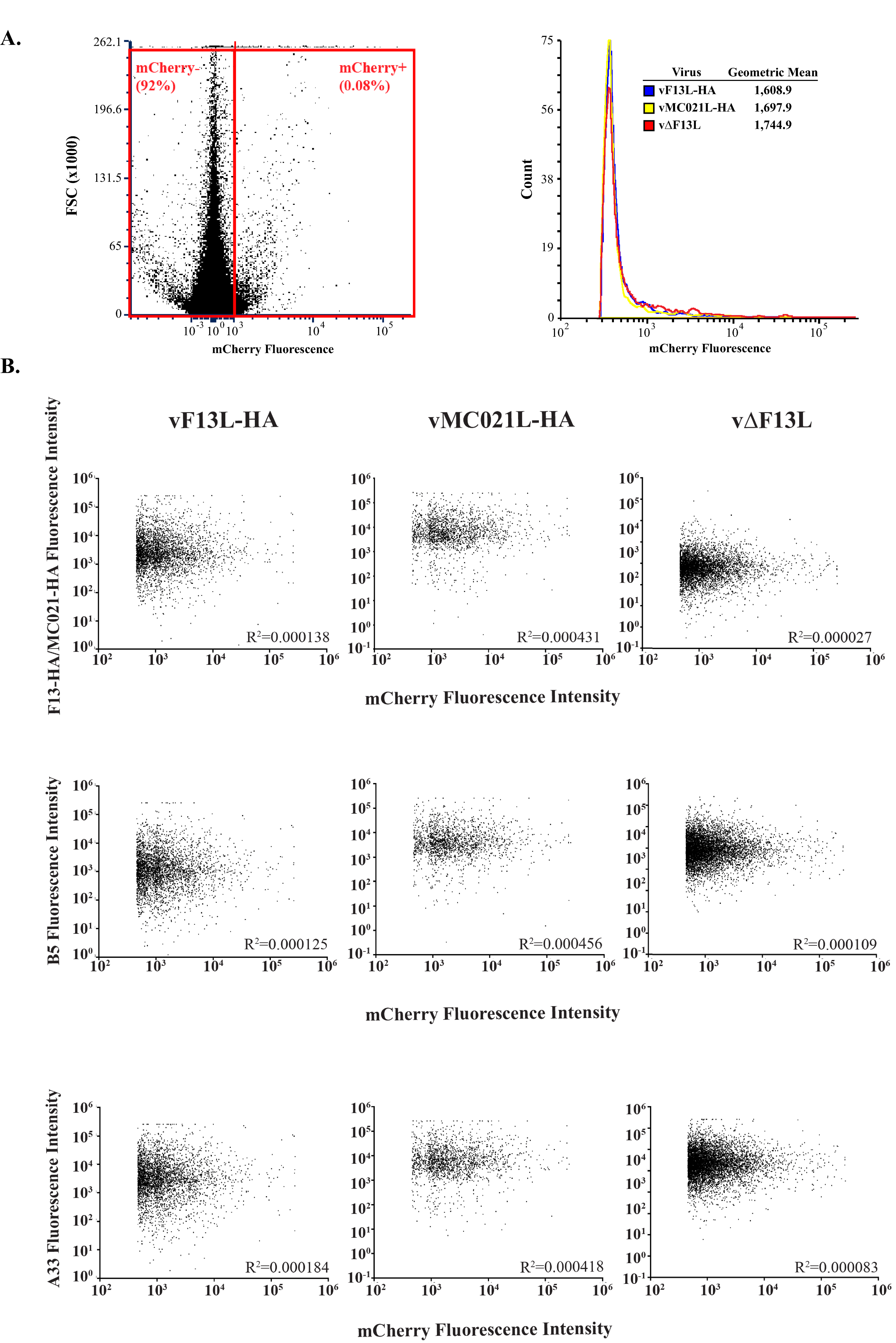

Supplement: S2 Fig — (A) RK13 cells were infected with vF13L-HA (left panel) or indicated viruses (right panel) at a MOI of 5 for 24 h. After, extracellular virions were purified as described, fixed, and analyzed by flow cytometry for mCherry fluorescence intensity. (Left Panel) Percentages represent the percent of events in each box relative to the total number of events both inside and outside of the boxes. (note some events fall outside of the red boxes). (Right Panel) Histogram showing the number of events at each mCherry fluorescence intensity for the indicated viruses. The calculated geometric mean for each virus is shown in the table. (B) The mCherry+ population, denoting virions, was analyzed for glycoprotein incorporation. Representative dot blots for the incorporation of mCherry and F13-HA/MC021-HA (rows; top), mCherry and A33 (rows; middle), and mCherry and B5 (rows; bottom) on viral surfaces for EV produced from vF13L-HA (columns; left), vMC021L-HA (columns; middle), and vΔF13L (columns right). (TIF) [file ppat.1010177.s002.tif]

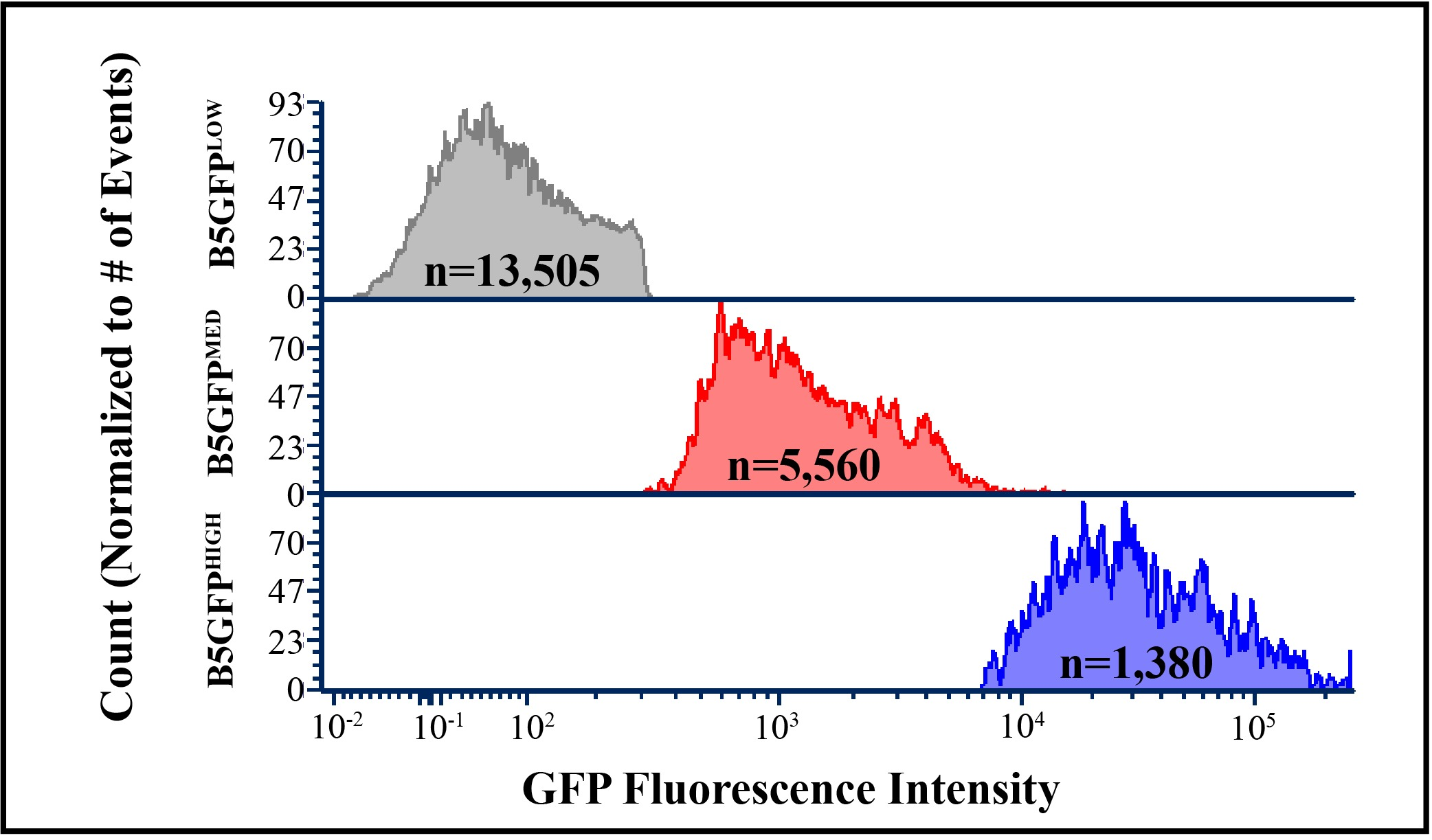

Supplement: S3 Fig — RK13 cells were infected with vF13L-HA/B5R-GFP at a MOI of 5 for 24 h. Extracellular virions were centrifuged through a sucrose cushion and analyzed by fluorescence-activated virion sorting. Virions were first gated on mCherry fluorescence intensity as in S1 Fig and mCherry+ events were subsequently sorted based on GFP fluorescence intensity (B5GFPHIGH, B5GFPMED, and B5GFPLOW). Y-axis is count normalized for the number of sorted events (n) for easier visualization, and n is denoted for each population. (TIF) [file ppat.1010177.s003.tif]
