# Supplementary material for: An increase in glycoprotein concentration on extracellular virions dramatically alters vaccinia virus infectivity and pathogenesis without impacting immunogenicity
Source: PLoS Pathog. 2021 Dec 28;17(12):e1010177. doi: 10.1371/journal.ppat.1010177 (PMC8746760; doi:10.1371/journal.ppat.1010177)
Supplement: S1 Table — Data from Fig 3B was used to generate dot plots of the detected signal for each particle analyzed. The resulting plots were analyzed for linear correlation (R2) using Pearson correlation coefficient. a ****, p<0.001. (DOCX) [file ppat.1010177.s004.docx]

S1 Table. Correlations of detected signal for B5-GFP recombinant viruses.

| **Virus** | **Protein** | **Pearson Correlation Coefficient (R^2^)** | **Significance^a^** |
| --- | --- | --- | --- |
| **vF13L-HA/B5R-GFP** | A33 vs. B5 | 0.8041 | **** |
|  | B5 vs. HA | 0.8750 | **** |
|  | A33 vs. HA | 0.7856 | **** |
| **vMC021L-HA/B5R-GFP** | A33 vs. B5 | 0.7005 | **** |
|  | B5 vs. HA | 0.6150 | **** |
|  | A33 vs. HA | 0.6993 | **** |
| **vΔF13L/B5R-GFP** | A33 vs. B5 | 0.9434 | **** |
|  | B5 vs. HA | 0.2186 | N/A |
|  | A33 vs. HA | 0.2560 | N/A |
